# Nanostructured lipid carriers for MicroRNA delivery in tumor gene therapy

**DOI:** 10.1186/s12935-018-0596-x

**Published:** 2018-07-13

**Authors:** Hairong Wang, Shiming Liu, Li Jia, Fengyun Chu, Ya Zhou, Zhixu He, Mengmeng Guo, Chao Chen, Lin Xu

**Affiliations:** 1 Special Key Laboratory of Gene Detection & Therapy of Guizhou Province, Zunyi, 563000 Guizhou China; 2 Department of Immunology, Zunyi Medical College, Zunyi, 563000 Guizhou People’s Republic of China; 3 Department of Medical Physics, Zunyi Medical College, Zunyi, 563000 Guizhou China; 4 Stem Cell and Tissue Engineering Research Center, Guizhou Medical University, Guiyang, 550004 Guizhou China

**Keywords:** Nanostructured lipid carrier (NLC), Tumor, MicroRNA

## Abstract

MicroRNAs (miRNAs), which are endogenous about 20–23 nucleotides non-coding RNAs, have been acted as post-transcriptional regulators of gene expression. Current studies demonstrated that miRNAs are promising candidates for tumor gene therapy because of their important biological functions in tumor cell proliferation, metastasis, apoptosis, and drug resistance. As an important delivery system, nanostructured lipid carriers (NLCs) have great potential in tumor gene therapy, particularly for miRNA delivery, due to low toxicity, low immunogenicity, long metabolic cycles, and easy modification. This article reviews recent research progress on NLCs for miRNA delivery in tumor gene therapy and prospective applications.

## Background

MicroRNAs (miRNAs), discovered in 1993, are double-stranded non-coding RNAs composed of about 20 nucleotides that regulate gene expression at the post-transcriptional level [1–4]. Extensive research works pointed out many miRNAs played important roles in tumor development, such as tumor cell proliferation, migration, apoptosis and drug resistance [5–8], therefore, miRNAs have been gradually applied in new therapeutic strategies for tumors [9]. However, because of low stability, low penetrability of cell membrane, and tissue non-specificity of miRNAs, researchers have studied the optimal ways to deliver these miRNAs into cells with different delivery system including nanostructured lipid carriers and have achieved significant progress [10, 11].

**Fig. 1:**
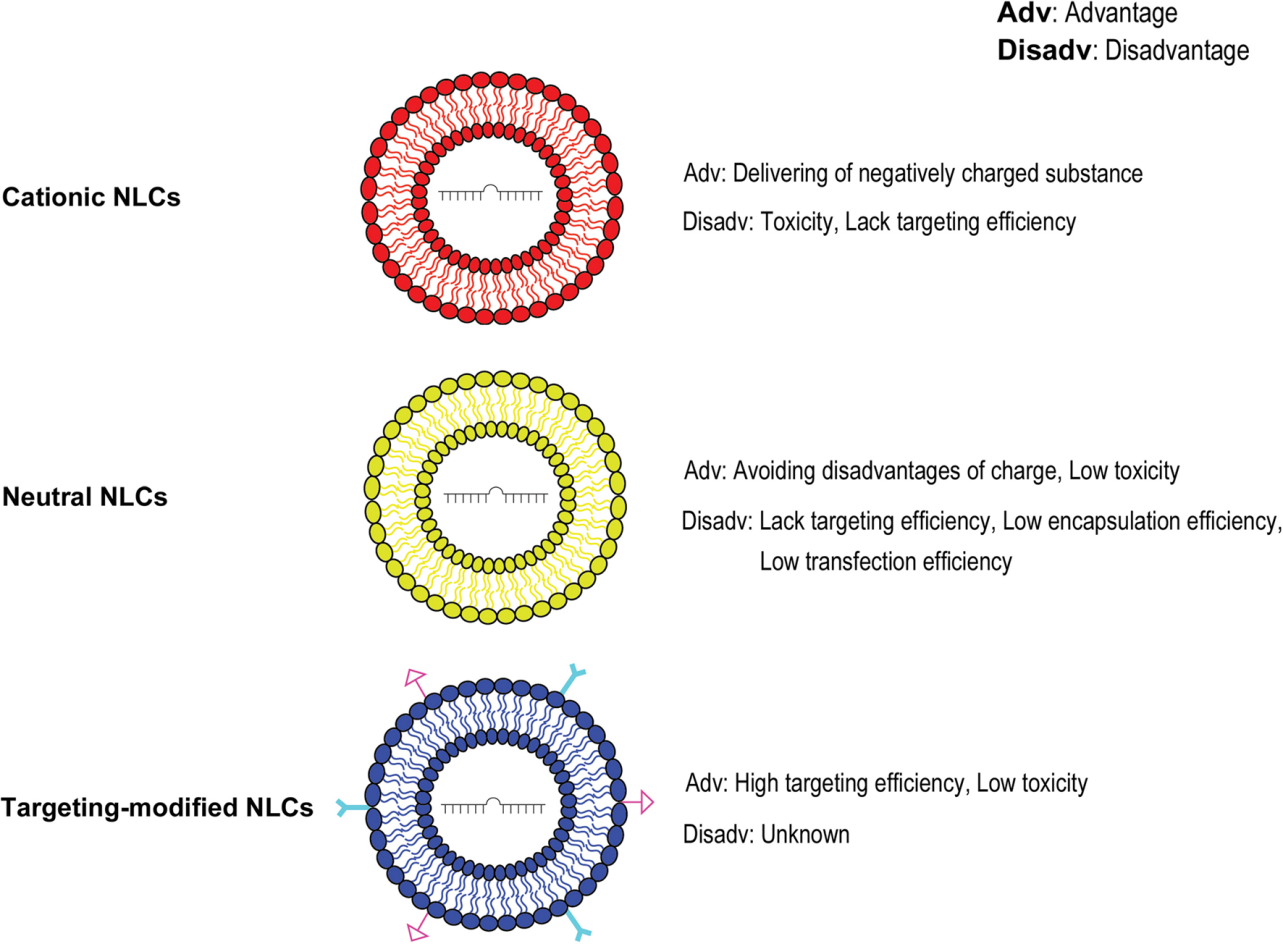
Current major types of nanostructured lipid carriers. The sketch of structure and characters of three major types of nanostructured lipid carriers (NLC): cationic NLC, neutral NLC, and targeting-modified NLC

Nanostructured lipids (NLs), first prepared in 1961 based on spherical vesicles by Bangham, are composed of a phospholipid bilayer with a diameter of tens to hundreds of nanometers [12–14]. NLs are the first nanoparticles applied in clinical medical research and widely used to deliver a variety of small molecules, chemical and biological drugs [15–17]. Recently, NLs have been improved to become nanostructured lipid carriers (NLCs), which have spherical structures with a mixed solid and liquid matrix, having an aqueous core surrounded by a lipid bilayer. NLCs have better entrapment efficiency, loading efficiency, and stability [18, 19]. Currently, there are three major types of NLC: cationic NLC, neutral NLC, and targeting-modified NLC (Fig. 1). Moreover, these types of NLCs have been widely used in the delivery of nucleic acids including distinct miRNA molecules for tumor gene therapy and have bright prospect for many clinical applications because of superior biocompatibility, high biodegradability and low immunogenicity [20–25].

## Cationic NLCs and delivery of miRNAs

Cationic NLCs are positively charged lipid vesicles and can be used as carriers for negatively charged substance, including proteins, polypeptides, oligonucleotides, RNAs and DNAs. Most cationic NLCs molecules are composed of three regions: a cationic head, a hydrophobic hydrocarbon backbone and a linker region. And cationic NLCs can improve the miRNA delivery efficiency as the result of electric charge interactions. For instance, Chen et al. [26] reported that cationic NLCs have been successfully used to deliver miR-34a to treat experimental lung metastasis of murine B16F10 melanoma. After treatment, tumor cell migration was significantly inhibited in vivo, indicating that cationic NLCs have potential for delivery of miRNAs in vivo. Furthermore, cationic NLCs have also been used to deliver miR-107 in the treatment of head and neck squamous cell carcinoma (HNSCC) in vitro and in vivo. Piao et al. [27] found that cationic NLCs could deliver miR-107 into tumor cells, and the clonogenic survival, cell invasion and cell migration of HNSCC cells were suppressed in delivery of miR-107 by cationic NLCs group compared with those in free miR-107 group, accompanied by decreased expression of tumor growth-related factor, such as protein kinase Cε (PKCε), cyclin-dependent kinase 6 (CDK6) and hypoxia-inducible factor 1-β (HIF1-β). In vivo experiment further revealed that in a preclinical mouse model of HNSCC, systemic administration of miR-107 delivered by cationic NLCs evidently stunted tumor growth by 45.2% compared to control group. These studies demonstrated the effectiveness of cationic NLCs for delivery of distinct miRNAs in cancer gene therapy. To cancer cells with drug resistance, recent evidence also suggested the efficacy of NLCs delivery system. For example, Rai et al. [28] conducted an in vivo study of delivery of miR-7-expressing plasmid by cationic NLCs to treat mouse xenograft model of human lung cancer. Their results showed that overexpression of miR-7 dramatically reversed the resistance effect of epithelial growth factor receptor-tyrosine kinase inhibitors (EGFR-TKIs) in lung cancer cells, suggesting the potential for use of cationic NLCs to deliver specific miRNAs to overcome tumor cell anti-cancer drug resistance [29]. Interestingly, Pramanik et al. [30] evaluated the safety of using Cationic NLCs delivery miR-34a or MiR-143/145 to treat pancreatic cancer xenograft model. In intravenous injection, cationic NLCs delivery miR-34a or MiR-143/145 effectively inhibited the growth of pancreatic cancer subcutaneous xenografts. Meanwhile, there were no obvious histopathologic changes or biochemical toxicity in mice, indicating safety of Cationic NLCs applied in vivo.

Recent studies have focused on the feasibility of cationic NLCs for delivery of miRNAs in combination therapy. For instance, Xu et al. [31] described the effect of the cationic NLCs were used to deliver miR-101 in combination with doxorubicin (DOX) to treat hepatocarcinoma xenograft model. They found that the proliferation, migration and invasion of hepatocarcinoma cells were inhibited obviously in co-delivery system miR-101/DOX group in vitro and in vivo. Notably, they further found that there were no significant difference in body weight of model mice between the combined treatment group and single treatment group. Taken together, their finding suggested that cationic NLCs did not increase the toxicity in combination therapy of tumors. Consistently, other studies also demonstrated that the effect of miRNAs in co-delivery system with cationic NLCs, including significantly improved chemotherapeutic sensitivity of tumor cells and the enhanced final therapeutic effects in combination therapy [32, 33]. For example, Yang et al. [34] found that cationic NLCs delivery of miRNA-375 effectively inhibited the proliferation, and enhanced the cell apoptosis as well as cell cycle arrest of cancer cells induced by cisplatin in combination with cisplatin in the treatment of hepatocellular carcinoma (HCC). Interestingly, this study also found that miRNA-375 in combination with cisplatin to treat the double oncogenes Akt/Ras-induced primary HCC mouse model could significantly delay the recurrence of tumor, indicating that the effect mechanism seems to be associated with the inhibition of the Akt signaling pathway by miR-375.

Over the years, cationic NLCs have been used as the standard carriers of RNA [35]. Nevertheless, cationic NLCs are also associated with significant toxicity problem. For instance, a high concentration of cationic NLCs could compromise the membrane integrity resulting in cell lysis and necrotic death. Moreover, at a sublethal concentration, cationic NLCs could still cause irritation to the cells and induce cell shrinkage, vacuolization of the cytoplasm and a reduced number of mitoses [36, 37].

## Neutral NLCs and delivery of miRNAs

In recent years, neutral NLCs have attracted attention as a novel carrier of miRNAs. Neutral NLCs have a targeted characteristic for delivery of miRNAs in vivo. Unlike cationic NLCs, neutral NLCs does not consist of cationic lipids. Thus, neutral NLCs can avoid a number of the disadvantages that can be attributed to charge. For example, neutral NLCs are not easy to form aggregates in biofluids and then avoid being filtered by the liver, adhering the endothelium or taken up by macrophages [38]. For instance, Trang et al. [4] reported that delivery of miR-124 by neutral NLCs via tail-vein injection has been used to treat mouse model of lung cancer. After 10 min of injection of miR-124/neutral NLC complex, the blood and three important organs including liver, kidneys and lungs of model mice have been analyzed. Data showed that both the blood and these important organs were elevated on the level of miR-124. To further confirm whether the miR-124 was uptaken by cells or existed only in the blood of the tissues, these organs of mice were perfused with 0.9% saline before analysis. It is worth noting that following perfusion with 0.9% saline, the level of miR-124 was decreased by 70–80% in liver and kidneys, indicating that the most of miR-124/neutral NLCs complex have effectively remained in the blood. However, saline perfusion rarely affected the level of miR-124 in the lungs, indicating that miR-124 was significantly taken up by lung tissue and neutral NLCs may be a useful vehicle to deliver distinct miRNAs to lung tumors.

In addition, neutral NLCs may have less toxicity than cationic NLCs. For example, Wiggins et al. [39] found that systemic delivery of miR-34a mimics used neutral NLCs has the potential to be translated into the lung cancer clinic. Their data showed the antioncogenic effects are accompanied by an accumulation of miR-34a in the tumor tissue and downregulation of direct miR-34a targets. Importantly, intravenous delivery of formulated miR-34a did not induce an elevation of cytokines or liver and kidney enzymes in serum, suggesting that the neutral NLCs is well tolerated and does not induce an immune response. Other studies further showed that tumor suppressor genes let-7 delivered by neutral NLCs in treatment of mouse models of lung cancer, not only preferred to target lung cancer cells, but also elicited no specific immune response in vivo [4]. Consistently, some reports also pointed out that delivery of miR-34a and miR-495 by neutral NLCs have been used to treat mouse model of diffuse large B cell lymphoma and lung cancer and could achieve good therapeutic effects. Meanwhile, these model mice were without serious adverse reactions [40, 41].

These results suggested the low toxicity of neutral NLCs applied in vivo. However, neutral NLCs were reported not only may reduce the RNA encapsulation efficiency, but also may decrease the transfection efficiency and subsequently the therapeutic effectiveness [22].

## Targeting-modified NLCs and delivery of miRNAs

Although delivery of miRNAs used both cationic NLCs and neutral NLCs was feasible, the target efficacy of these delivery systems was still lacking in vivo. Therefore, targeting-modified NLCs have been a focus of current research works. To reduce the recognition and phagocytosis of NLCs by macrophages, NLCs could be coated with biocompatible polymers. Such as polyethylene glycol (PEG), which significantly improved stability and half-life of NLCs in vivo. Moreover, to increase the concentrations of miRNA/NLC complex in target tissues, ligands of specific cells could be conjugated to the surface of the NLC, which improved targeting of NLC delivery [42]. In addition, NLCs have also been modified by different ligands might have different biological features, such as life-span, targeting ability and cellular permeability [25]. For example, Hayward et al. [43] reported that miRNA-125-a-5p was delivered by NLCs in which the surface was coated with Hyaluronic Acid (HA) was used to treat HER2 positive metastatic breast cancer. Their results showed that miRNA-125-a-5p primarily targeted metastatic breast cancer cells (21MT-1) which were isolated from the metastatic pleural effusion over normal breast tissue via an intrinsic HA-CD44 mediated endocytosis, had the ability to escape from the intracellular endolysosomal pathway to effectively induced gene silencing, subsequently could knock out HER2 proto-oncogenes which was involved in both transcriptional and translational regulations. Meanwhile, the related pathways including the PI3K/Akt and MAPK signaling pathways, cell proliferation as well as cell migration, which were also significantly inhibited. Similarly, Chen et al. [26] reported that delivery of miR-34a by NLCs modified with tumor-targeting single-chain variable fragment (scFv) has been used to treat experimental lung metastasis of murine B16F10 melanoma. The experimental results showed that miR-34a was delivered by the scFv targeted NLCs efficiently induced tumor cell apoptosis and inhibited tumor cell migration in vivo, which was associated with the downregulation of survivin and the inhibition of the MAPK pathway. In terms of ligands, for instance, Zhang et al. [44] conducted a study in which transferrin (Tf) modified NLCs was used to deliver miR-221 antisense oligonucleotides (anti-miR-221) to the human Hepatocellular carcinoma (HCC) cell line HepG2. Their data showed that the average size of these particles was 122.5 nm and the encapsulation efficiency of about 70%. Moreover, the Tf modified NLCs was most stable at 4 °C. Importantly, their experiment data showed that Tf modified NLCs were able to deliver anti-miR-221 more efficiently and thus provided better efficacy than nontargeted NLCs in the HepG2 cells through the Tf-mediated endocytosis process.

Recently, other studies further found that the effectiveness of delivery of targeting-modified NLCs was related to particle size and encapsulation efficiency. For example, Lee et al. [45] documented that ephrin-A1 (ephrin type-A receptor 1) modified NLCs have also been used to deliver let-7-a into mouse models of non-small cell lung cancer (NSCLC). Data showed that the ephrin-A1 modified NLCs with an average diameter of 100 nm showed high stability, low cytotoxicity, and high loading efficiency of let-7a and ephrin-A1. Moreover, ephrin-A1 modified NLCs could inhibit NSCLC proliferation, migration and tumor growth, as well as could improve the effectiveness of targeted delivery of let-7a. However, the correlation among the delivery efficiency of targeting-modified NLCs, particle size and encapsulation efficiency, as well as size limitation of protein fragments, especially, the relationship between delivery efficiency and local temperature of tumors in vivo remain to be elucidated in the future.

It would be noticed that in recent studies the combination of targeting-modified NLCs delivery miRNA and other drugs have also achieved progress. Costa et al. [46] documented that chlorotoxin (CTX) targeted NLCs have been used to deliver anti-miR-21 for treatment of glioblastoma (GBM). Their data showed that the encapsulation efficiency of anti-miR-21 was above 85% and a mean particle size was less than 190 nm. Meanwhile, intravenously-administration of anti-miR-21 delivered by the CTX targeted NLCs led to preferential accumulation within brain tumors, and without obvious systemic immune injury. Additional studies reported that systemic administration of anti-miR-21 delivered by CTX targeted NLCs in combination with sunitinib also efficiently inhibited tumor cell proliferation, promoted tumor cell apoptosis and increased the survival of GBM-bearing mice, indicating the potential prospect of targeting-modified NLCs delivery in combination therapy strategies.

## Summary

Recent studies have shown that NLCs had good prospective application as a new type of carriers for delivery of miRNAs in tumor gene therapy and combination therapy (Table 1). However, many questions remain unanswered, and require further study. For example, in the process of gene therapy in vivo, what are the relationships between NLCs particle size and effectiveness of targeting delivery? What is the size limitation of the protein fragments utilized in the targeting-modified NLCs in vivo? Furthermore, how to evaluate the relationship between the uptaken of NLCs by normal organs and tissues and the efficiency of miRNA was delivered by NLCs in the condition of distinct delivery routes? Also, what is the pharmacokinetics of NLCs in vivo? And so on.

**Table 1:** NLC delivery for miRNA in tumor gene therapy

Nanocarriers	miRNA	Tumor therapy	Organ uptake
Cationic NLC	miR-34a	B16F10 melanoma lung metastasis [26]	N/A
	miR-107	Head and neck squamous carcinoma [27]	N/A
	miR-7	Lung cancer [28, 29]	N/A
	miR-34a/miR-143/miR-145	Pancreatic carcinoma [30]	N/A
	miR-101	Hepatocarcinoma [31]	N/A
	miR-375	Hepatocellular carcinoma [34]	Liver tissue, kidney tissue
Neutral NLC	miR-124	Lung cancer [4]	Lung tissue
	let-7	Lung cancer [4]	N/A
	miR-34a	Lung cancer [39]	N/A
	miR-34a	Diffuse large B cell lymphoma [40]	N/A
	miR-495	Lung cancer [41]	N/A
Targeting-modified NLC	miRNA-125a-5p	Breast cancer [43]	N/A
	miR-34a	B16F10 melanoma lung metastasis [26]	N/A
	anti-miR-221	Hepatocarcinoma [44]	N/A
	let-7a	NSCLC [45]	N/A
	anti-miR-21	Glioblastoma [46]	Brain tissue

*NLC* nanostructured lipid carrier, *NSCLC* non small cell lung cancer, *N/A* not applicable

In all, taking into account in-depth studies of NLCs regarding molecular structures, releasing mechanisms, and pharmacokinetics in vivo, and along with a better understanding of biological functions and tumorigenesis mechanisms of miRNAs, tumor gene therapy with NLC-delivered miRNAs will become safer, more effective and stable, thereby promoting the development of clinical novel cancer therapeutic strategies.

## Abbreviations

miRNAs: microRNAs
NLCs: nanostructured lipid carriers
NLs: nanostructured lipids
